# C3H10T1/2 Mesenchymal Stem Cell Line as a New In Vitro Tool for Studying Adipocyte Dedifferentiation

**DOI:** 10.3390/biology14040444

**Published:** 2025-04-20

**Authors:** Yuriko Yuuki, Takeshi Katafuchi, Tomohiko Kazama, Taro Matsumoto, Makoto Makishima

**Affiliations:** 1 Division of Biochemistry, Department of Biomedical Sciences, Nihon University School of Medicine, Itabashi-ku, Tokyo 173-8610, Japan; yu-yuuki@tky.ndu.ac.jp (Y.Y.); makishima.makoto@nihon-u.ac.jp (M.M.); 2 Division of Cell Regeneration and Transplantation, Department of Functional Morphology, Nihon University School of Medicine, Itabashi-ku, Tokyo 173-8610, Japan; kazama.tomohiko@nihon-u.ac.jp (T.K.); matsumoto.taro@nihon-u.ac.jp (T.M.)

**Keywords:** C3H10T1/2, mesenchymal stem cell, dedifferentiated fat cell, adipogenesis, osteogenesis, chondrogenesis

## Abstract

Dedifferentiated fat (DFAT) cells are adipocyte-derived cells that are able to differentiate into multiple cell lineages such as adipocytes, osteoblasts and chondrocytes, similar to mesenchymal stem cells (MSCs). Despite their great potential for developing novel clinical interventions by using their multipotency, the detailed mechanisms of how adipocytes undergo dedifferentiation into DFAT cells are not completely understood, because useful in vitro tools for studying adipocyte dedifferentiation are missing. In this study, we show that mature adipocytes derived from the MSC cell line C3H10T1/2 underwent dedifferentiation into cells with DFAT cell-like characteristics, when they were cultured in an inverted flask. During the dedifferentiation, expression levels of genes and protein specific to adipocytes were continuously decreased, whereas those for MSC, proliferation and WNT/β-catenin signaling were gradually increased. These DFAT-like cells also underwent differentiation into adipocytes, osteoblasts and chondrocytes with their specific cell morphology and gene expression. We also observed that an individually cultured single adipocyte also underwent dedifferentiation into DFAT-like cells that were able to differentiate into the multiple cell lineages. Our results indicate that C3H10T1/2 cells could be a great tool for determining molecular biological and biochemical mechanisms underlying adipocyte dedifferentiation.

## Simple Summary

The aim of this study is to establish a live tissue-free method to study adipocyte dedifferentiation using adipocytes derived from C3H10T1/2, a commonly used mesenchymal stem cell line. These in vitro adipocytes were able to undergo dedifferentiation simply via performing a series of collagenase treatments, centrifugation and culturing in an inverted cell culture-coated flask filled completely with a regular cell culture medium. The dedifferentiated adipocytes were morphologically similar to the original undifferentiated C3H10T1/2 cells accompanied with the reduced expression levels of genes specific to adipogenesis and induced expression levels of those specific to preadipocytes and proliferation. The dedifferentiated cells were further confirmed to carry potential to differentiate into adipocytes, osteoblasts and chondrocytes, which were verified with the corresponding staining and quantification of marker protein and gene levels.

## 1. Introduction

Adipocytes are the major cellular component of adipose tissues (ATs) containing large lipid droplets, in which triglycerides are stored as a source of energy [1]. Adipocytes are derived mainly from mesenchymal stem cells (MSCs) that are located in the bone marrow, with a potential to differentiate into other multiple cell lineages including osteoblasts and chondrocytes [2]. Accumulating evidence have shown that mature adipocytes conversely undergo dedifferentiation into MSC-like multipotent cells. Yagi et al. isolated mature adipocytes from mouse fat pads by collagenase digestion, cultured them in an inverted culture flask filled completely with a growth medium, called ceiling culture, and found the appearance of cells with fibroblast-like morphology, designated dedifferentiated fat (DFAT) cells [3]. DFAT cells are capable of self-renewal and differentiation into adipocytes in a culture dish and even in mouse tissue. They have been further investigated regarding their multipotency, and it has been shown that both DFAT cells derived from mouse subcutaneous adipocytes and bone marrow adipocytes undergo adipogenesis, osteogenesis and chondrogenesis [4]. Moreover, Matsumoto et al. succeeded in obtaining DFAT cells from human adipose tissues [5] and demonstrated that their surface antigen profiles and their potential to differentiate into adipocytes, osteoblasts and chondrocytes almost meet the minimum criteria for defining multipotent MSCs [6].

Several studies have been performed in this decade using DFAT cells to develop new stem cell therapies. For example, DFAT cells have been shown to induce angiogenesis in a mouse ischemia tissue, revealing that DFAT cells could be a useful tool for inducing neovascularization in damaged tissue [7]. Transplantation of polyglycolic acid nerve conduits filled with DFAT cells significantly promoted nerve regeneration in rat facial tissues with nerve defects [8]. Jumabay et al. developed DFAT cells both from mouse and human brown adipocytes and also succeeded in inducing neurogenic differentiation in vitro [9]. In addition to these observations, implantation of DFAT cells showed a positive therapeutic effect on diseases such as glomerulonephritis [10,11], inflammatory bowel disease [12] and cerebral infarction [13] in animal models. One of the greatest advantage of DFAT cells is that they can be induced from the ATs of patients themselves without any potentially harmful gene manipulations, which are necessary for making induced pluripotent stem cells [14,15]. It is therefore critical to clarify the detailed machineries of the dedifferentiation for development of a more efficient method to obtain DFAT cells and to also prevent the occurrence of potentially deleterious secondary responses. However, we have only a limited amount of data to understand the molecular biological and biochemical background of what triggers adipocytes to undergo dedifferentiation and how they actually undergo dedifferentiation into DFAT cells.

C3H10T1/2 cells are a well-characterized MSC cell line that was originally established from embryos of the C3H mouse strain [16]. Since the discovery of the multipotency of this cell line to differentiate into adipocytes, osteoblasts and chondrocytes, this cell line has been utilized as an excellent model of MSCs to characterize how MSCs can differentiate into those cells [17,18,19]. We therefore ask if mature adipocytes derived from C3H10T1/2 cells (C3H10T1/2 adipocytes) undergo dedifferentiation into cells that share the MSC-like differentiation potency with DFAT cells from mature adipocytes in ATs. We, in fact, found the appearance of multiple cells lacking visible lipid droplets, hereafter termed DFAT-like cells, from C3H10T/2 adipocytes. In this study, we demonstrate that C3H10T1/2 adipocytes could be a useful tool for seeking the dedifferentiation mechanism from adipocytes to DFAT cells.

## 2. Materials and Methods

### 2.1. Cell Culture

C3H10T1/2 cells were obtained from the Riken Cell Bank (Tsukuba, Japan), and monolayer cultures were maintained in 10 cm culture plates in a culture medium comprising low-glucose Dulbecco’s Modified Eagle Medium (L-DMEM, Fujifilm Wako, Osaka, Japan) supplemented with 10% heat-inactivated fetal bovine serum (FBS) and 1× antibiotic mixture (100 U/mL penicillin, 100 μg/mL streptomycin and 250 ng/mL Amphotericin B; Fujifilm Wako). The cells were incubated in a humidified incubator at 37 °C and 5% CO_2_. The medium was changed every 2–3 days, and the cells were passaged before reaching confluence.

### 2.2. Adipogenesis

The cells were harvested by trypsinization and seeded onto 10 cm plates or multi-well plates at a cell density of 3.5 × 10^4^ cell/cm^2^. On the following day, the culture medium was replaced with an adipogenesis induction medium comprising high-glucose DMEM (H-DMEM, Fujifilm Wako) containing the 1× antibiotic mixture, 10% FBS, 5 μg/mL bovine insulin (Sigma-Aldrich, St. Louis, MO, USA), 1 μM dexamethasone (Sigma-Aldrich) and 50 μg/mL indomethacin (Nacalai Tesque, Kyoto, Japan), as reported by Lehman et al. with minor modifications [20]. The cells were then cultured for 7 days. The cells were further cultured with H-DMEM containing 1× antibiotic mixture, 10% FBS and 1 μg/mL bovine insulin for the next 7 days. The medium was replaced every 2–3 days.

### 2.3. Ceiling Culture

**Figure 1:**
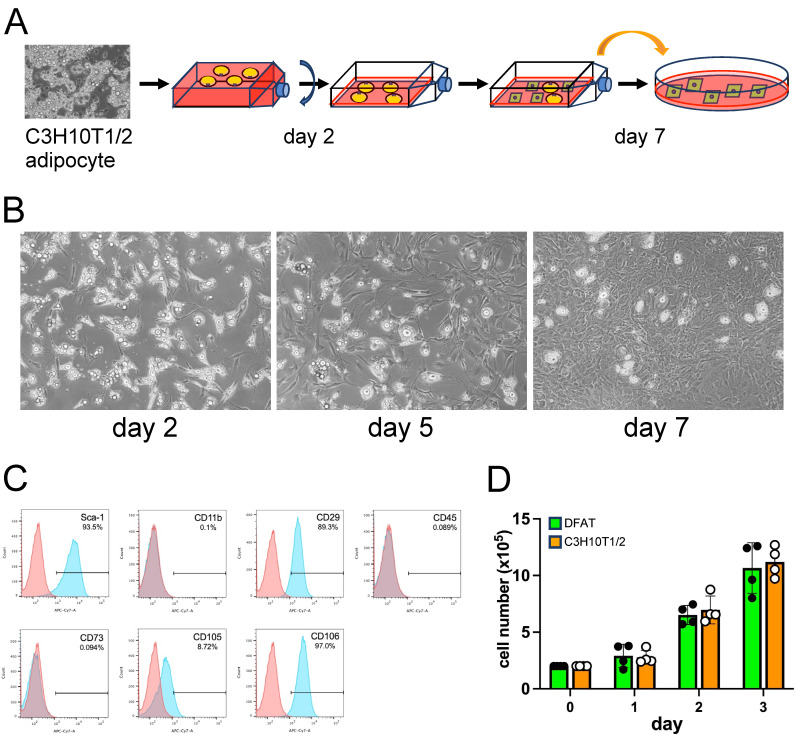
C3H10T1/2 adipocytes underwent dedifferentiation into DFAT-like cells. (**A**) C3H10T1/2 adipocytes were detached from culture plates by collagenase treatment, separated from undifferentiated cells by centrifugation and cultured in an inverted flask for the first two days. Then, the culture medium was replaced with fresh medium, and the cells were further cultured in the same flask in the regular upright orientation. On day 7, the cells in the flask were treated with trypsin and centrifuged, and the cells of the bottom fraction were seeded onto a regular culture plate. (**B**) DFAT-like cells derived from C3H10T1/2 adipocytes. Cells carrying lipid droplets were attached to the adhesive surface of an inverted culture flask on day 2, and the appearance of the DFAT-like cells became apparent on day 5. The DFAT-like cells finally reached semi-confluence on day 7. Scale bar: 200 μm. (**C**) Flow cytometry analysis for characterization of the DFAT-like cells. (**D**) Proliferation of the DFAT-like cells (DFAT) and C3H10T1/2 parental cells. Values are expressed as mean ± SE; significant differences among cell numbers at different time points for the DFAT-like cells and C3H10T1/2 cells were determined by one-way ANOVA and Tukey’s post hoc test, and those between the DFAT-like cells and C3H10T1/2 cells at the same time points were determined by Student’s *t*-test.

Ceiling culture was performed as described by Matsumoto et al. [5] with minor modifications, as shown in Figure 1A. C3H10T1/2 adipocytes on 10 cm plates were incubated with collagenase II solution containing 1 mg/mL collagenase II (Worthington Biochemical Corporation, Lakewood, NJ, USA) and 1% bovine serum albumin (Fujifilm Wako) in Hank’s balanced salt solution containing Ca^2+^ and Mg^2+^ (Fujifilm Wako) at 37 °C for 30 min in a humidified incubator with occasional rocking. The cells were then peeled off by pipetting, and the cell suspension was centrifuged at 200× *g* for 3 min. The adipocytes in the floating top layer were transferred to a tube filled with a fresh culture medium, suspended and centrifuged for washing. The adipocytes in the top layer were washed again by resuspending them with a fresh medium and centrifuged. Following the second round of cell wash, the adipocytes in the top layer were transferred to a new tube. Then, the adipocyte number was counted, and 5 × 10^5^ adipocytes were transferred to a 25 cm^2^ flask filled completely with the culture medium. The flask was subsequently turned upside down for ceiling culture, which allowed adipocytes, with their specific gravity smaller than that of the culture medium, to attach to tissue culture-treated surface of the flask. The cells were incubated in a humidified incubator at 37 °C and 5% CO_2_. Two days later, the medium was replaced with fresh medium (5 mL), and the flask was rotated to the usual upright orientation. The cells were further cultured for five more days until they reached semi-confluence.

### 2.4. Flow Cytometry

Flow cytometric analysis was performed to characterize both the DFAT-like cells and undifferentiated C3H10T1/2 cells. The following antibodies were used in the MSC marker antibody panel (R&D Systems, Minneapolis, MN, USA): anti-Sca1-APC, anti-CD11b-APC, anti-CD29-APC, anti-CD45-APC, anti-CD73-APC, anti-CD105-APC and anti-CD106-APC. The cells were analyzed with an FACS Aria flow cytometer using Cell Quest Pro software, version 5.1 (Becton Dickinson, Franklin Lakes, NJ, USA) [7]. The number of positive cells was compared with the signal obtained for the corresponding immunoglobulin isotypes.

### 2.5. Oil Red O Staining

The cells were rinsed once with PBS and fixed with 4% formaldehyde in PBS at room temperature for 15 min. Then, the cells were rinsed once with PBS, twice with H_2_O and twice with 60% 2-propanol. Staining was performed using 0.18% Oil Red O (Sigma-Aldrich) in 60% 2-propanol at 37 °C for 30 min, and the cells were rinsed once with 60% 2-propanol and twice with H_2_O. After the cell images were photographed with a Nikon Eclipse inverted microscope Ts2R (Tokyo, Japan), the Oil Red O that had adsorbed to the cells were recovered by incubation with 100% 2-propanol for 30 min at room temperature. The OD was determined at 510 nm by spectrophotometry. To normalize the staining levels using the cellular DNA content, the cells were suspended with SNET buffer (10 mM Tris (pH 8), 100 mM NaCl, 10 mM EDTA, 0.5% SDS) containing 10 μg/mL RNase A and incubated at 65 °C for 1h, which was followed by proteinase K digestion (20 μg/mL) at 65 °C overnight. The DNA was purified using phenol/chloroform extraction, and following ethanol precipitation, the amount was quantified through measurement of the OD at 260 nm.

### 2.6. Osteogenesis and Alizarin Red S Staining

The cells were seeded on 12-well plates at the cell density described above and cultured overnight. The medium was replaced with osteogenesis induction medium comprising L-DMEM containing an antibiotic mixture, 10% FBS, 10 nM dexamethasone, 50 μg/mL ascorbate (Sigma-Aldrich) and 5 mM glycerol-2-phosphate (Sigma-Aldrich), and the cells were cultured for 28 days as described by Alonso-Perez et al. with minor modifications [21]. The medium was replaced every 3–4 days. Mineral nodule formation was observed from Alizarin Red S (Sigma-Aldrich) staining. The cells were fixed as described above, rinsed once with PBS and twice with H_2_O, and incubated with Alizarin Red S solution (pH 4.8, Sigma-Aldrich) at room temperature for 30 min. The cells were washed three times with H_2_O and photographed. The Alizarin Red S that had adsorbed onto the cells was recovered by incubation with 10% acetic acid for 30 min at room temperature, and the OD was determined at 450 nm by spectrophotometry. The staining intensity was normalized using the cellular DNA content as described.

### 2.7. Chondrogenesis and Alcian Blue Staining

Following trypsinization, the cells were suspended with Ham’s F-12 medium (Nacalai Tesque) containing the antibiotics mixture and 10% heat-inactivated FBS (F-12 medium) and transferred to a 15 mL tube. Following centrifugation at 200× *g* for 3 min, the cells were resuspended with F-12 medium at a concentration 10^7^ cell/mL. A total of 10 μL of the cell suspension was placed in a 24-well tissue culture dish to make a small round spot at the center of the wells for micromass culture, and the cells were allowed to attach to the bottom of the wells at 37 °C in a 5% CO_2_ humidified incubator for 3h. Then, Ham’s F-12 medium containing 100 ng/mL human bone morphogenetic protein-2, 40 μg/mL proline and 50 μg/mL ascorbate was added to the wells. The cells were further cultured for 14 days, with medium replacements every 2–3 days, as reported by Denker et al. with minor modifications [18]. For Alcian Blue staining, the micromass cultures were fixed as described above, washed twice with PBS and twice with H_2_O, and then incubated with 0.1% Alcian Blue 8GX (Abcam, Cambridge, UK), pH 1.0, at room temperature for 30 min. The cells were washed three times with H_2_O and photographed. The Alcian Blue that had adsorbed onto the cells was recovered by incubation with 6 M guanidium-HCl for 6 h at room temperature. The OD was determined at 630 nm by spectrophotometry [22]. The staining intensity was normalized using the cellular DNA content as described.

### 2.8. Real Time Polymerase Chain Reaction

RNAs were purified from the cells using RNAiso Plus reagent (Takara Bio, Shiga, Japan). A total of 1 μg of total RNA was reverse-transcribed using Improm II reverse transcriptase (Promega, Madison, WI, USA) with random hexamers according to the manufacturer’s instructions. The gene expression levels in the cells were quantified by real-time polymerase chain reaction (RT-PCR) using Power SYBR Green PCR Master Mix (Thermo Fisher Scientific, Waltham, MA, USA) and the primer sets listed in Table 1. Relative mRNA levels were calculated using the comparative Ct method, normalized to the mRNA level of *U36b4*.

### 2.9. Western Blotting

The cells were solubilized in SDS-PAGE buffer (2% SDS, 5% glycerol and 62.5 mM Tris (pH 6.8)), and the amount of protein was quantified using Pierce BCA Protein Assays (Thermo Fisher Scientific). Then, 10 μg or 30 μg of the solubilized protein mixture was mixed with bromophenol blue and 2-mercaptoethanol to make the final concentrations of 0.01% and 2%, respectively, boiled for 5 min, and electrophoresed on SDS-PAGE gel. The resolved proteins were transferred to a nitrocellulose membrane using a Trans-Blot semi-dry transfer system (Bio-Rad, Tokyo, Japan). Immobilized proteins were blocked with 5% nonfat dry milk in TBS-T (150 mM NaCl, 10 mM Tris pH 7.4 and 0.1% Tween 20) at room temperature for 30 min and then incubated at 4 °C with a primary antibody dissolved with 5% bovine serum albumin in TBS-T. The following primary antibodies were used to probe proteins of interest: anti-FABP4 (Elabscience, cat. #E-AB-52150), anti-ITGA5 (Bioassay Technology Laboratory, cat. #BT-AP03159), anti-CCND1 (Assay Genie, cat. #CAB19038), anti-OPN (Bioassay Technology Laboratory, cat. #BT-AP06578), anti-SOX9 (Merck, cat. #MABC785) and anti-GAPDH (Cell Signaling Technology, Danvers, MA, USA, cat. #97166). Then, the membranes were incubated at room temperature with a horse radish peroxidase-conjugated anti-rabbit (Cell Signaling Technology, cat. #7074) or anti-mouse (Cell Signaling Technology, cat. #7076) secondary antibody in TBS-T. The membranes were finally incubated at room temperature with chemiluminescence solution containing 100 mM Tris pH 8.5, 0.2 mM p-coumaric acid, 1.25 mM luminol and 0.0915% H_2_O_2_. The proteins of interest were visualized using a Fusion chemiluminescent image detection system (Vilber-Lourmat, Collégien, France), and the band intensities were quantified using Image J software version 1.52a.

### 2.10. Statistical Analysis

All the statistical analyses were performed using GraphPad Prism 10. The values in the figures correspond to the mean ± standard error of the mean (SE) of at least three samples. Student’s *t*-test was used to assess differences between two groups. One-way analysis of variance (ANOVA), followed by Tukey’s post hoc test, was used to assess statistical differences between more than three groups unless otherwise stated. *p*-values less than 0.05 were considered statistically significant.

## 3. Results

### 3.1. C3H10T1/2 Adipocytes Underwent Dedifferentiation

The fully differentiated adipocytes derived from the C3H10T1/2 cells were harvested and transferred to an inverted flask and cultured (Figure 1A). Two days later, a large number of adipocytes were already attached to the flask with projections of pseudopodia (Figure 1B). Then, we re-inverted the flask to the upright orientation, replaced the medium and cultured the attached adipocytes. On day 5, the appearance of cells lacking visible lipid droplets with fibroblast-like morphology became apparent (Figure 1C), and they finally reached semi-confluence on day 7 (Figure 1B). We termed those fibroblast-like cells “DFAT-like” cells and analyzed their surface antigen profiles. These DFAT-like cells exhibited a surface antigen profile similar to that of DFAT cells prepared from mouse adipose tissues [7], expressing high levels of Sca-1, CD29 and CD106, a moderate level of CD105, and undetectable levels of CD11b, CD45 and CD73 (Figure 1C). The surface antigen profile of the DFAT-like cells was almost identical to that of undifferentiated C3H10T1/2 cells (Figure S1), revealing that DFAT-like cells maintain an MSC-like surface antigen profile even after the adipogenesis–dedifferentiation cycle. DFAT-like cells also maintain their proliferation potential with the growth rate, similar to that of parental C3H10T1/2 cells (Figure 1D).

To evaluate what percent of undifferentiated C3H10T1/2 cells were contaminated in this adipocyte preparation, we took a small fraction of cells in the top adipocyte layer. The adipocytes were then double-stained with Hoechst 33342 and Bodipy 493/503 to stain cell nuclei and lipid droplets, respectively, and observed under a microscope. However, a significant number of Bodipy 493/503-positive cells lacked Hoechst 33342-positive nuclei, probably because they somehow lost the nuclei during the preparation. We therefore took another small fraction of adipocytes and seeded them into a slide chamber. The side chamber was subsequently filled completely with the culture medium, covered with a small piece of adhesive Aeroseal cell culture film, inverted, and placed in the cell culture incubator for 24 h. The attached cells were double-stained again with Hoechst 33342 and Bodipy 493/503 (Figure S2). We observed that only five cells were Bodipy 493/503-negative out of 327 Hoechst 33342-positive cells (Figure S2D), revealing that 98.5% of the attached cells were adipocytes. However, there is a possibility that even these five cells were derived from adipocytes and had already lost their lipid droplets due to earlier dedifferentiation during the 24 h culture.

### 3.2. Gene Expression Levels During Dedifferentiation

**Figure 2:**
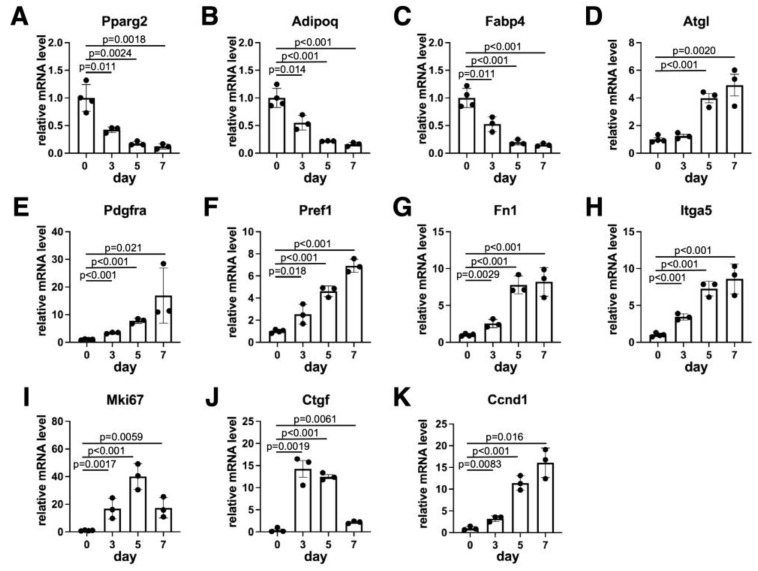
Temporal pattern of gene expressions. The expression levels of marker genes for adipocytes (**A**–**D**), preadipocytes (**E**–**H**), proliferation (**I**) and WNT/β-catenin signaling (**J**,**K**). Values are expressed as mean ± SE; significant differences between relative mRNA levels on day 0 and other days were determined by one-way ANOVA and Dunnett’s post hoc test. N = 3–4.

We next examined the temporal pattern of expression for several adipocyte markers that were considered to be expressed during the dedifferentiation process. As expected, the expression levels of adipocyte-specific genes, such as *Pparg2, Adipoq* and *Fabp4*, and the protein levels of FABP4 were the highest before starting the ceiling culture and gradually reduced (Figure 2A–C and Figure S3). The expression level of *Atgl*, which is an adipocyte-specific lipase and critical for adipocyte dedifferentiation [23,24], gradually increased (Figure 2D) during the dedifferentiation. We next evaluated the expression levels of MSC markers. We found that *Pdgfra*, *Pref1* and *Fn1* were gradually elevated and reached the highest levels on day 7 (Figure 2E–G). Similarly, both the expression levels of *Itga5* and protein levels of ITGA5 were also gradually elevated (Figure 2H and Figure S3). These data are consistent with the observation in Figure 1C that adipocytes were gradually replaced with DFAT-like cells. The expression level of *Mki67*, a proliferation marker gene, was elevated during dedifferentiation. We also observed the increased levels of WNT/β-catenin signaling marker genes such as *Ctgf* and *Ccnd1* during dedifferentiation (Figure 2I–K). Our Western blotting data confirmed that CCND1 protein levels were also increased, and its temporal pattern was similar to that of ITGA5 (Figure S3). *Mki67* and *Ctgf* levels reached the maximum on day 5 and day 3, respectively, and slightly decreased on day 7, probably due to their contact inhibition.

### 3.3. DFAT-like Cells Were Able to Differentiate into Multiple Cell Lineages

**Figure 3:**
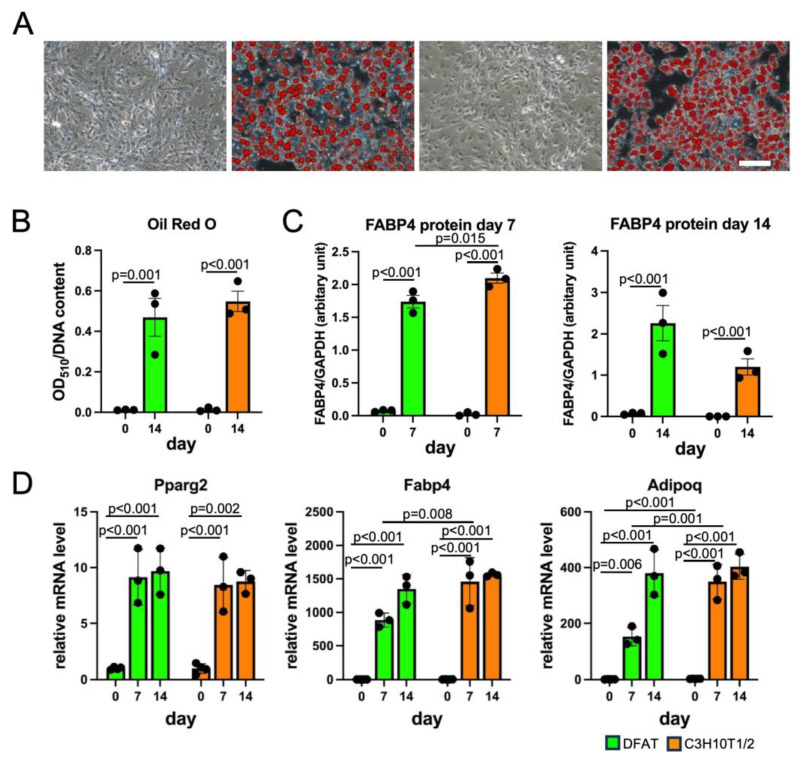
Adipogenic differentiation of DFAT-like cells. (**A**) Oil Red O staining of adipocytes derived from DFAT-like cells (left) and C3H10T1/2 cells (right). Cells were cultured with adipogenic medium for 28 days and then stained with Oil Red O. Scale bars: 200 μm. (**B**) Quantification of Oil Red O staining. (**C**) Protein levels of FABP4. (**D**) Expression levels of *Pparg2*, *Fabp4* and *Adipoq*. Values are expressed as mean ± SE; significant differences among relative mRNA or protein levels at different time points in DFAT-like cells and C3H10T1/2 cells were determined by one-way ANOVA and Tukey’s post hoc test. N = 3.

**Figure 4:**
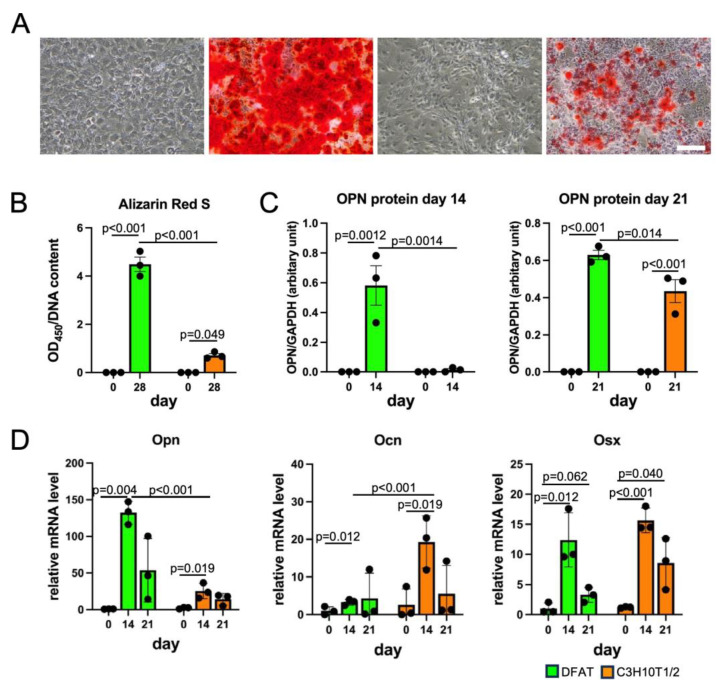
Osteogenic differentiation of DFAT-like cells. (**A**) Alizarin Red S staining of osteoblasts derived from DFAT-like cells (left) and C3H10T1/2 cells (right). Cells were cultured with an osteogenic medium for 28 days and then stained with Alizarin Red S. Scale bars: 200 μm. (**B**) Quantification of Alizarin Red S staining. (**C**) Protein levels of OPN. (**D**) Expression levels of *Opn*, *Ocn* and *Osx*. Values are expressed as mean ± SE; statistical significance was determined as described in the legend of Figure 3. N = 3.

**Figure 5:**
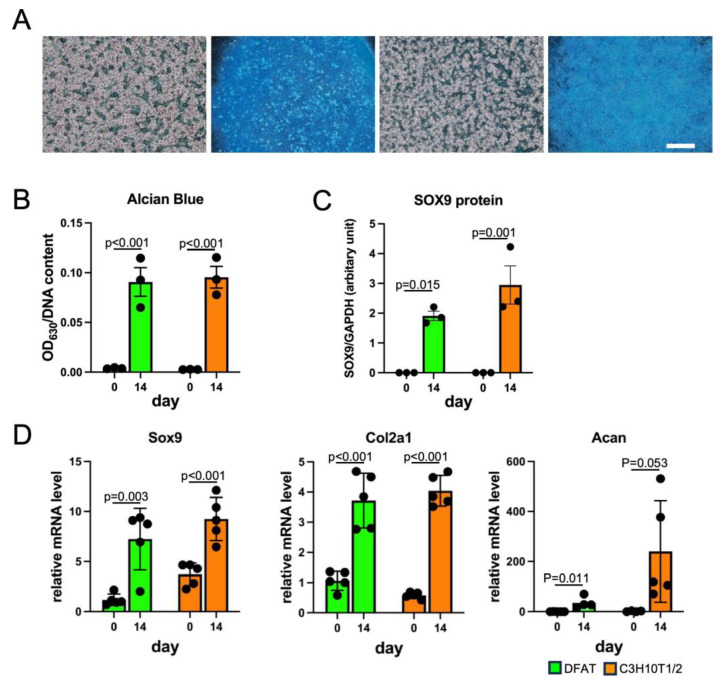
Chondrogenic differentiation of DFAT-like cells. (**A**) Alcian Blue staining of chondrocytes derived from DFAT-like cells (left) and C3H10T1/2 cells (right). Cells were cultured with chondrogenic medium for 14 days and then stained with Alcian Blue. Scale bars: 500 μm. (**B**) Quantification of Alcian Blue staining. (**C**) Protein levels of SOX9. (**D**) Expression levels of *Sox9*, *Col2a1* and *Acan*. Values are expressed as mean ± SE; statistical significance was determined as described in the legend of Figure 3. N = 3.

We next investigated if DFAT-like cells undergo differentiation into adipocytes, osteoblasts and chondrocytes and subsequently compared their MSC-like potency with that of C3H10T1/2 cells. We first cultured DFAT-like cells with an adipogenic medium to test if they re-differentiate into adipocytes. As shown in Figure 3A,B, DFAT-like cells formed large Oil Red O-stained lipid droplets after 14-day adipogenic induction, and the staining level was comparable to that of C3H10T1/2 cells. The protein levels of FABP4 in both cells were almost undetectable on day 0 and also elevated after 7-day and 14-day adipogenic induction, despite the slightly lower levels of FABP4 protein in DFAT-like cells than C3H10T1/2 cells on day 7. (Figure 3C and Figure S4A). The gene expression levels of *Pparg2*, *Adipoq* and *Fabp4* were induced in DFAT-like cells and C3H10T1/2 cells, despite significantly lower levels of *Adipoq* and *Fabp4* mRNAs on days 0 and 7 in DFAT-like cells (Figure 3D). We next cultured DFAT-like cells and C3H10T1/2 cells with osteogenic medium. Regarding their accumulation of calcium phosphate, we observed more intense staining with Alizarin Red S in DFAT-like cells after 28-day osteogenic induction (Figure 4A,B). We also observed an elevation in OPN protein levels after 14-day and 21-day osteogenic induction (Figure 4C and Figure S4B), and the OPN protein levels in DFAT-like cells were significantly higher than those in C3H10T1/2 cells. The transcription levels of osteoblast marker genes were also evaluated, and we detected higher levels of *Opn* and lower levels of *Ocn* in osteogenic cells derived from DFAT-like cells than those in C3H10T1/2 cells on 14 day, whereas no significant difference was observed in the levels of *Osx* (Figure 4D). We finally performed micromass culture on the cells [18] and found that both DFAT-like cells and C3H10T1/2 cells treated with chondrogenic medium for 14 days were stained more intensely with Alcian Blue (Figure 5A,B) than those before the treatment. However, the staining levels of both cells were comparable to each other (Figure 5B). The SOX9 protein levels were higher in both DFAT-like cells and C3H10T1/2 cells after 14-day chondrogenic induction, and the magnitude of their protein levels was comparable to each other (Figure 5C and Figure S4C). We also evaluated the expression levels of chondrocyte marker genes and observed that *Sox9* and *Col2a1* levels were significantly higher and *Acan* levels tended to be elevated in the cells treated with the chondrogenic medium than in the pretreated cells. However, we did not find any significant difference in the levels of those genes between DFAT-like cells and C3H10T1/2 cells, even before and after the treatment (Figure 5D). Collectively, our data reveal that C3H10T1/2 cells possessed MSC-like differentiation potency even after the adipogenesis–dedifferentiation cycle, despite this cycle slightly changing the differentiation potentials.

### 3.4. Individually Cultured Single Adipocytes Underwent Dedifferentiation to Proliferate DFAT-like Cells

**Figure 6:**
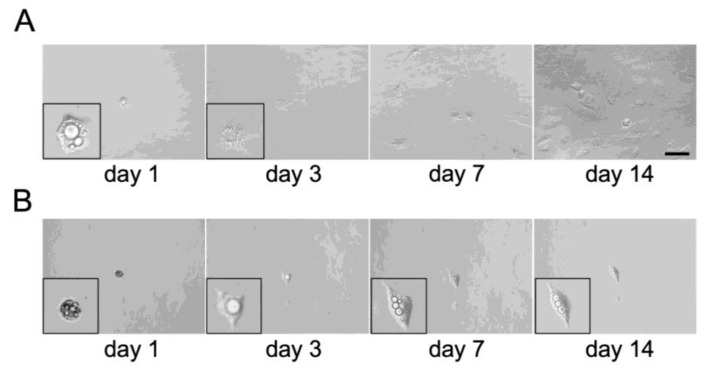
Representative images of dedifferentiation of a single C3H10T1/2 adipocyte. Temporal pattern of an individually cultured single adipocyte dedifferentiating into proliferative DFAT-like cells (**A**) and the one which failed dedifferentiation (**B**). Inset pictures show the cell magnified. Scale bar: 100 μm.

We observed multiple undifferentiated cells even after adipogenic induction for 14 days (Figure 1A) and five cells lacking visible lipid droplets after 24 h of ceiling culture (Figure S2). It could be reasonable to believe that all the adipocytes, osteoblasts and chondrocytes observed in Figure 3, Figure 4 and Figure 5 were simply derived from those undifferentiated cells but not from DFAT-like cells. To rule out this possibility, we attempted to obtain DFAT-like cells of single-adipocyte origin and analyze their multipotency. To carry this out, we first dispensed a medium containing approximately 30 adipocytes into a 96-well plate, which allowed each adipocyte to be randomly distributed to one of these wells. We then filled each well completely with a growth medium and performed ceiling culture. We found 18 bottoms of wells in which only one adipocyte was attached after 24 h ceiling culture. We then observed that 12 adipocytes had actually lost their lipid droplets, changed their morphology to become similar to DFAT-like cells, proliferated, and finally reached semi-confluence on day 14 (Figure 6A). The other six cells exhibited a small morphological change without losing lipid droplets and did not proliferate at all (Figure 6B).

### 3.5. Single-Adipocyte-Derived DFAT-like Cells Were Also Able to Differentiate into Multiple Cell Lineages

**Figure 7:**
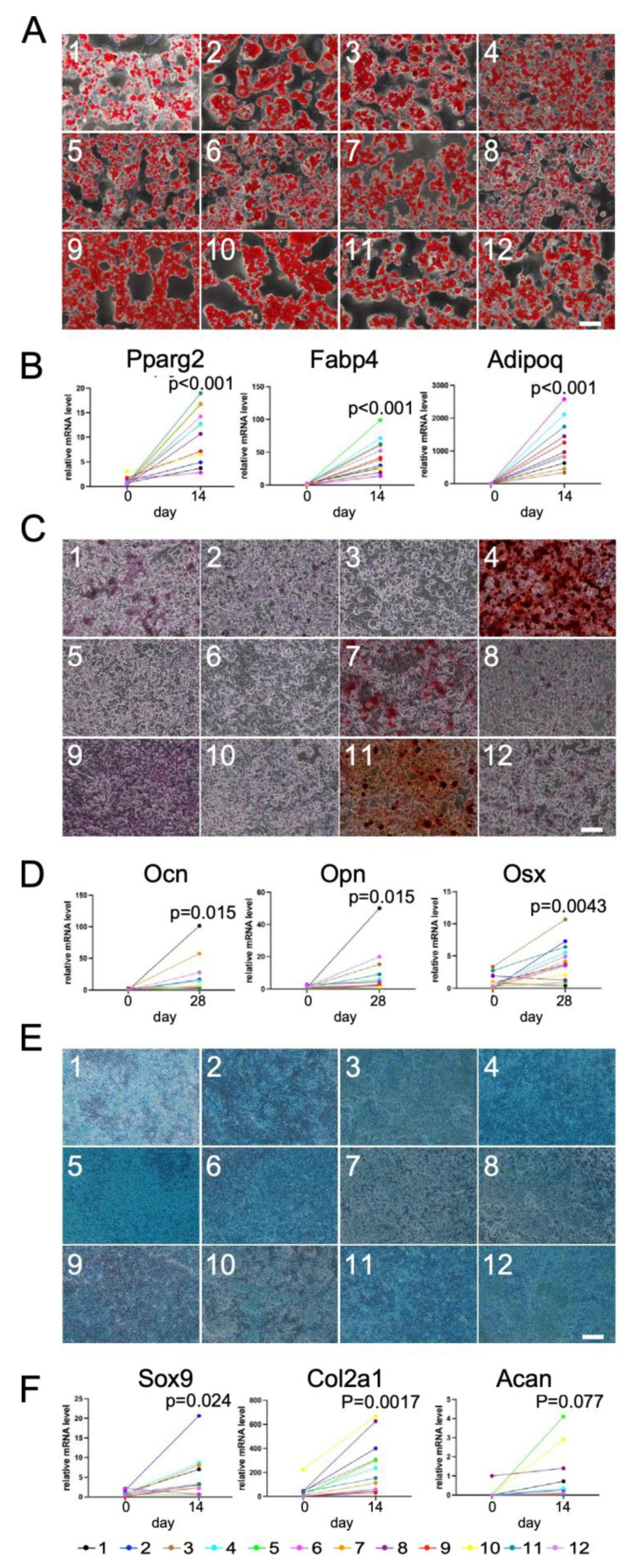
Adipogenic, osteogenic and chondrogenic differentiation of DFAT-like cells of 12 different cells of single-adipocyte origin. (**A**) Oil Red O staining of adipocytes derived from 12 DFAT-like cells of different single-adipocyte origin. (**B**) Expression levels of *Pparg2*, *Fabp4* and *Adipoq*. (**C**) Alizarin Red S staining. (**D**) Expression levels of *Opn*, *Ocn* and *Osx*. (**E**) Alcian Blue staining. (**F**) Expression levels of *Sox9*, *Col2a1* and *Acan*. Values are expressed as mean ± SE; significant differences between relative mRNA levels were determined by Student’s *t*-test. Scale bars: 200 μm (**A**,**C**) and 500 μm (**E**).

We next transferred these 12 single-adipocyte-derived DFAT-like cells into regular cell culture plates to culture them with adipogenic, osteogenic and chondrogenic media. As shown in Figure 7, all the 12 different lines of cells of different single-adipocyte origin exhibited potential of differentiating into adipocytes (Figure 7A,B) and chondrocytes (Figure 7E,F), and many of them exhibited potential of differentiating into osteoblasts (Figure 7C,D). These data reveal that a large fraction of C3H10T1/2 adipocytes were capable of dedifferentiating into cells with multipotency.

Despite almost all 12 cell lines exhibiting elevated mRNA levels of those marker genes, we observed those levels in the differentiated cells to be considerably varied. These variations gave rise to our idea that if we could find any significant correlations among the expression levels of those marker genes in the 12 differentiated cells of different single-adipocyte origin, we could take advantage of those variations. We first found that the expression levels of *Pparg2*, *Adipoq* and *Fabp4* in 12 different adipocytes exhibited significant positive correlation among all of them, revealing that PPARγ, a ligand-activated transcription factor (nuclear receptor) called the master regulator of adipogenesis, plays a critical role in regulation of *Adipoq* and *Fabp4* expression levels (Figure S5A–C). Among the three osteogenic genes, on the other hand, we found significant positive correlation only between the expression levels of *Opn* and *Ocn* (Figure S5D–F). Regarding chondrogenesis, we found a tendency toward positive correlation only between the expression levels of *Col2a1* and *Acan* (Figure S5I). The expression levels of *Sox9*, on the other hand, exhibited no correlation with the other two genes (Figure S5G,H). We also expected some negative correlations between adipogenic and osteogenic genes, adipogenic and chondrogenic genes, or osteogenic and chondrogenic genes. However, we did not detect any negative or even positive correlations among them at all.

## 4. Discussion

In this study, we developed a live tissue-free method for investigating adipocyte dedifferentiation using a commonly used MSC cell line, C3H10T1/2. The advantages of studying adipocyte dedifferentiation using culture cells are as follows: (1) ATs from live animals or human patients are not necessary as a source of adipocytes, and only cell culture facilities are required to study adipocyte dedifferentiation. (2) It is easy to expand the number of adipocytes. Any size of experiments is possible. (3) Only two days of ceiling culture and another five days of regular culture are enough to obtain dedifferentiated cells. (4) A relatively high percentage of C3H10T1/2 adipocytes undergo dedifferentiation. As shown in Figure 6 and Figure 7, 12 out of approximately 30 adipocytes were able to dedifferentiate into DFAT-like cells with multiple potency. (5) C3H10T1/2 cells are available in multiple major cell sources such as the Riken Cell Bank and American Tissue Culture Collection. In addition, it is commonly recognized that studying preadipocyte cell lines such as 3T3-L1 cells has greatly contributed to understanding the detailed mechanisms underlying adipogenesis, and in fact, numerous molecules critical to the regulation of adipocytes have been discovered by using this preadipocyte cell line [25,26,27,28]. We therefore expect that studying the adipocyte dedifferentiation of C3H10T1/2 cells would provide a variety of significant ways to unveil common molecular biological and biochemical mechanisms underlying adipocyte dedifferentiation.

Although both DFAT-like cells and their parental C3H10T1/2 cells exhibited similar surface antigen profiles and overall multipotency, we also detected some differences. For example, we observed a delayed elevation in *Fabp4* and *Adipoq* mRNA levels and FABP4 protein levels in DFAT-like cells (Figure 3). DFAT-like cells also exhibited more intense staining by Alizarin Red S (Figure 4A) after the treatment with osteogenic medium. They also showed higher *Opn* mRNA levels and OPN protein levels, revealing that they have significantly higher osteogenic potential than C3H10T1/2 cells (Figure 4B,C). We have no evidence to explain how DFAT-like cells acquire these differentiation potentials. Regarding the elevated Alizarin Red S staining in DFAT-like cells, however, Kishimoto et al. described observations similar to ours [29]. Using human buccal fat pads, they prepared DFAT cells from adipocytes to compare their osteogenic potential with that of adipocyte stem cells (ASCs) isolated from the same fat pads. They subsequently found that osteoblastic cells derived from DFAT cells were more strongly stained by Alizarin Red S than those from ASCs. These data suggest that dedifferentiation from adipocytes somehow facilitates cells in differentiating into osteoblastic cells. Further investigation is necessary to clarify how DFAT cells acquire higher osteogenic potential.

Adipocyte dedifferentiation occurs not only on a plastic plate; it has also been observed in live tissues as a physiological event by multiple independent groups. For example, mammary gland adipocytes undergo dedifferentiation during pregnancy and transform proliferative Pdgfrα^+^ fibroblast-like adipocyte precursor cells during lactation [30,31]. These dedifferentiated cells subsequently re-differentiate into adipocytes during involution. Adipocytes in the dermal adipose tissues also undergo dedifferentiation into their precursor cells for the maintenance of skin homeostasis. Zhang et al. first described the importance of the differentiation–dedifferentiation process of dermal adipocytes in the hair cycle and wound healing [32]. Adipocytes in the bone marrow can dedifferentiate into mesenchymal stromal cells, which potentially give rise to osteogenic lineage cells. Interestingly, this dedifferentiation process was impaired in adipocytes lacking *Atgl* [23,24], suggesting that triglycerides in adipocytes inhibit adipocyte dedifferentiation. We also observed that the expression level of *Atgl* was elevated after the onset of ceiling culture (Figure 2). In addition, we found in our single-adipocyte analysis that the cell lost lipid droplets before they started self-renewal, whereas the cells maintaining visible lipid droplets for 14 days did not proliferate (Figure 6). These facts corroborate the importance of the de-esterification of triglycerides in adipocyte dedifferentiation.

There have been a few attempts to clarify the molecular biological and biochemical mechanisms of adipocyte dedifferentiation of potential interest. Zoico et al. described that the dedifferentiation of 3T3-L1 adipocytes was induced when they were co-cultured with a pancreatic carcinoma cell line, MIA PaCa-2, or cultured with conditioned media, and showed that the inhibition of a WNT5a signal with an anti-WNT5 antibody inhibited dedifferentiation [33]. On the contrary, Li et al. demonstrated that the dedifferentiation of adipocytes was elevated by physical compression, whereby both cytosolic and nuclear β-Catenin levels were elevated using mouse adipocytes. They also observed that the dedifferentiation of adipocytes was activated by WNT3a, while it was reduced by IWP2, an inhibitor of endogenous WNT ligand secretion [34]. Similarly, adipocytes derived from 3T3-L1 cells or stromal vascular fraction of mouse inguinal ATs were cultured using a hypertonic medium, and it was found that the secretion of extracellular vesicles containing mitochondrial components from those cells was increased and stimulated TNF-α signaling to activate WNT/β-Catenin signaling to increase the dedifferentiation of those adipocytes [35]. Sun et al. described that the IL-1β/NF-kB/CREB signaling axis activates wound-induced adipogenesis, which was accompanied with the suppression of WNT/β-Catenin signaling in the early stages of the healing process of dermal wounds, whereas WNT signal activation by the addition of WNT3A, ablation of *Gsk3*, an inhibitory factor of WNT/β-Catenin signaling, or inhibition of GSK3β by lithium induced the dedifferentiation of dermal adipocytes to contribute to myofibroblast formation in the late stages of the healing process [36]. As shown in Figure 2 and Figure S3, we observed that adipocyte dedifferentiation was accompanied with the upregulation of *Ccnd1* and *Ctgf*, both of which are the target genes of WNT/β-catenin [37], revealing that our data are consistent with the observations of Sun et al. Either way, WNT/β-Catenin signaling is highly likely to be involved in the dedifferentiation of adipocyte in a context-dependent manner. Other interesting data regarding adipocyte dedifferentiation using a sophisticated ceiling culture chip have been described [38]. They showed that adipocyte dedifferentiation was regulated by extracellular matrices such as fibronectin, and also by intracellular remodeling of the structure of actin. They therefore identified that the Hippo, Hedgehog and PPARγ signaling pathways were potent regulators of adipocyte dedifferentiation. Consistent with their observation regarding PPARγ signaling, we observed that the expression levels of *Pparg2*, *Adipoq* and *Fabp4* were gradually reduced during dedifferentiation (Figure 2).

Our study has some significant limitations. First, since we did not transplant DFAT-like cells into animals such as C3H mice, we have no data as to whether DFAT-like cells are able to differentiate into tri-lineage cells in vivo, as tested by Tang et al. using parental C3H10T1/2 cells. [39]. In this study, we solely focused on the development of animal-free methods to study adipocyte dedifferentiation. Second, we did not identify what molecules trigger the initiation of the dedifferentiation process. We surmise that WNT/β-catenin signaling molecules are involved in the initiation of this process, but we have no evidence yet. Future studies could aim to address these limitations.

## 5. Conclusions

In this study, we showed data regarding the great potential of C3H10T1/2 cells from studying in vitro adipocyte dedifferentiation. C3H10T1/2 is a commonly used cell line that is easy to culture and induce adipogenesis in. This cell line could be a gold standard for studying in vitro adipocyte dedifferentiation and eventually clarifying its detailed mechanisms that are yet to be unveiled.

## Figures and Tables

**Table 1:** Primer sets for RT-PCR.

	Gene (Accession #)	Forward	Reverse
Reference	*U36b4* (NM_007475)	5′-cgtcctcgttggagtgaca-3′	5′-cggtgegtcagggattg-3′
Adipogenesis	*Pparg2* (NM_011146)	5′-caccagtgtgaattacagcaaatc-3′	5′-acaggagaatcteccagagtite-3′
	*Adipoq* (NM_009605)	5′-tgtcagtggatctgacgaca-3′	5′-aacaggagagcttgcaacagt-3′
	*Fabp4* (NM_024406)	5′-gtgaaaacttegatgattacatgaa-3′	5′-gcctgccactttecttgtg-3′
	*Atgl* (NM_025802)	5′-caacgccactcacatctacgg-3′	5′-ggacacctcaataatgttggcac-3′
Osteoblast	*Ocn* (NM_001305448)	5′-ccaagcaggagggcaata-3′	5′-tegtcacaagcagggtca-3′
	*Osx* (NM_130458)	5′-gggagcagagtgccaaga-3′	5′-tactectggcgcataggg-3′
	*Opn* (NM_001204201)	5′-ttgcttttgcctgtttggca-3′	5′-gatctgggtgcaggctgtaa-3′
	*Sox9* (NM_011448)	5′-gagcccgatctgaagaagga-3′	5′-gcttgacgtgeggcttgttc-3′
Chondrocyte	*Col2a1* (NM_031163)	5′-caccaaattcctgtteagcc-3′	5′-tgcacgaaacacactggtaag-3′
	*Acan* (NM_001361500)	5′-aacaactgcaggctgcctat-3′	5′-ccagggaactegtccttgtc-3′
Preadipocyte	*Pdgfra* (NM_001083316)	5′-ctcacagacttcggaagaga-3′	5′-aagtcgctctcacacactta-3′
	*Pref1* (NM_010052)	5′-gacctggagaaaggccagta-3′	5′-agggagaaccattgatcacg-3′
	*Fn1* (NM_010233)	5′-gccacacctacaaccagtat-3′	5′-gggctggaaagattactctc-3′
	*Itga5* (NM_010577)	5′-cctagccattctttttggcc-3′	5′-ggcttgagctgagctttttc-3′
Proliferation	*Mki67* (NM_001081117)	5′-tcattgaccgctcctttaggt-3′	5′-ttgaccttccccatcagggt-3′
WNT/β-catenin	*Ctgf* (NM_010217)	5′-ccgccaaccgcaagat-3′	5′-cgacccaccgaagacaca-3′
	*Ccnd1* (NM_001379248)	5′-caacttcctctcctgctaccg-3′	5′-ccttgtttagccagaggccg-3′

## Data Availability

The data of the present study are available upon reasonable request from the corresponding author.

## Supplementary Materials

The following supporting information can be downloaded at https://www.mdpi.com/article/10.3390/biology14040444/s1, Figure S1: Flow cytometry analysis for characterization of undifferentiated C3H10T1/2 cells; Figure S2: Visualization of cells lacking lipid droplets in a slide chamber after ceiling culture for 24 h. Figure S3: Temporal pattern of protein levels of FABP4, ITGA5 and CCND1. Figure S4: Protein levels of FABP4, OPN and SOX9. Figure S5: Correlations among the expression levels of adipogenic marker genes, osteogenic marker genes and chondrogenic marker genes of differentiated DFAT-like cells of single-adipocyte origin.
