# Supplementary material for: C3H10T1/2 Mesenchymal Stem Cell Line as a New In Vitro Tool for Studying Adipocyte Dedifferentiation
Source: Biology (Basel). 2025 Apr 20;14(4):444. doi: 10.3390/biology14040444 (PMC12024763; doi:10.3390/biology14040444)
Supplement: Supplementary file 1 [file biology-14-00444-s001.zip › biology-3554224-supplementary.pdf]

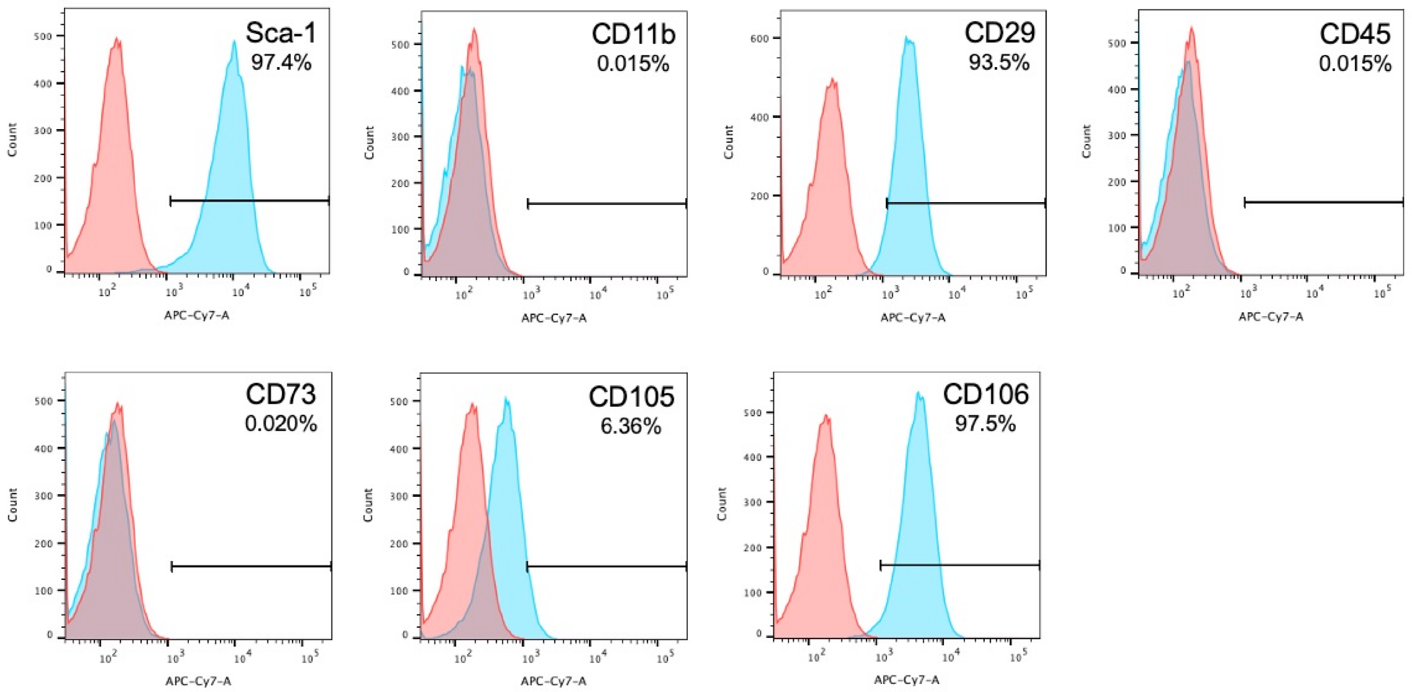


**Figure S1. Flow cytometry analysis for characterization of undifferentiated C3H10T1/2 cells.**

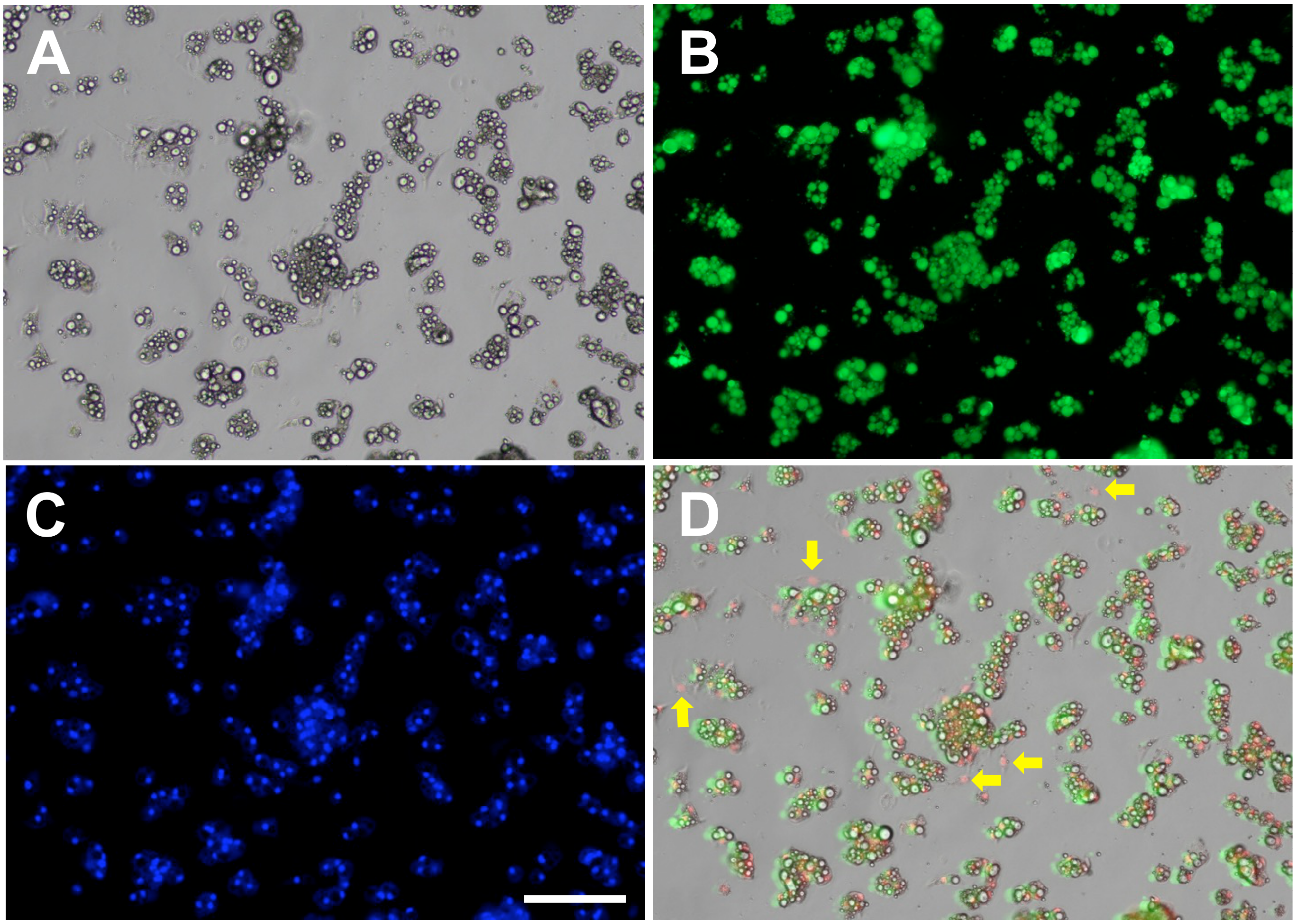


**Figure S2. Visualization of cells lacking lipid droplets in a slide chamber after ceiling culture for 24h.** Phase contrast image of adipocytes (A), nuclei stained with Hoechst 33342 (B), lipid droplets stained with Bodipy 493/503 (C) and all the three images were overlayed using Image J software (D). Arrows indicate the cells lacking visible lipid droplets. Note that the color of nuclei was switched to red in the overlayed image to facilitate visualization. Scale bar: 500 μm.

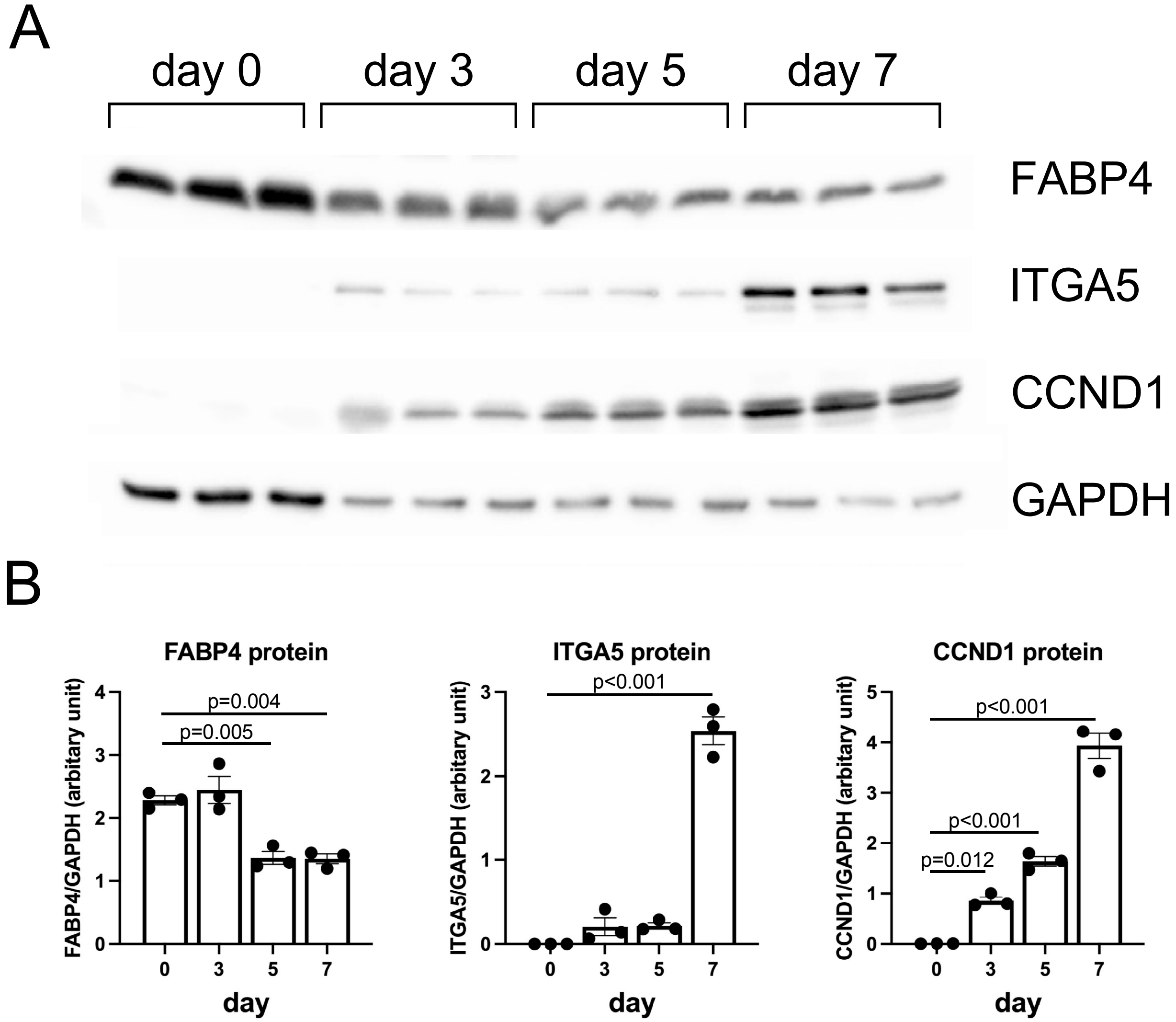


**Figure S3. Temporal pattern of protein levels of FABP4 (adipocyte marker), ITGA5 (preadipocyte marker), CCND1 (WNT/b-catenin signaling marker) and GAPDH (loading control) during adipocyte dedifferentiation.** (A) Western blot images. Ten mg of protein extract was loaded in each lane. (B) Quantification of FABP4, ITGA5 and CCND1 protein levels normalized with that of GAPDH. Values are mean ± SE; significant differences among protein levels of different time points were determined by one-way ANOVA and Dunnett's post hoc test.

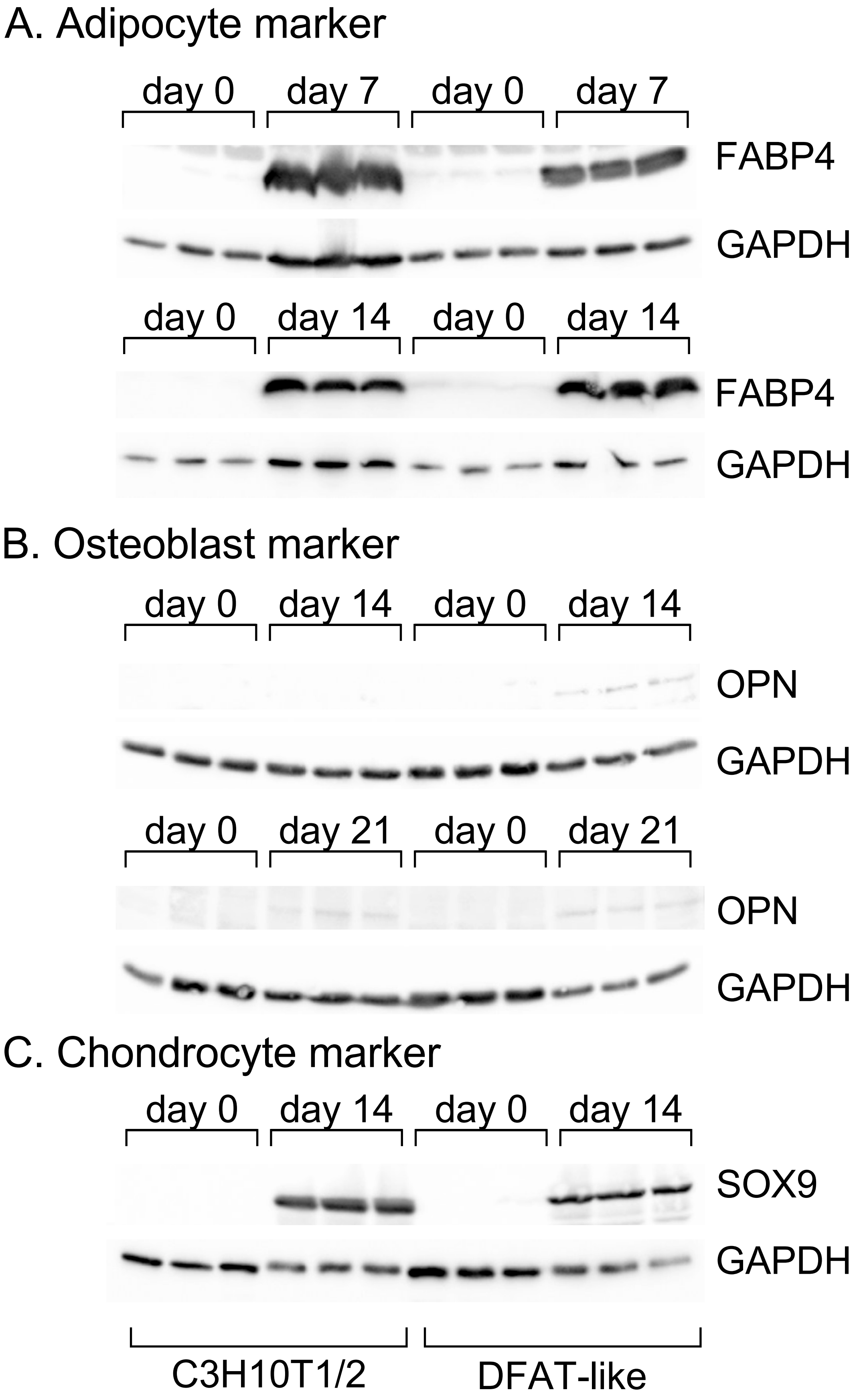


**Figure S4. Protein levels of FABP4 (adipocyte marker), OPN (osteoblast marker), SOX9 (condrocyte marker) and GAPDH (loading control).** Cells were treated with the differentiation media for the indicated days. Ten μg (A, C) or 30 μg (B) of protein was loaded in each lane.

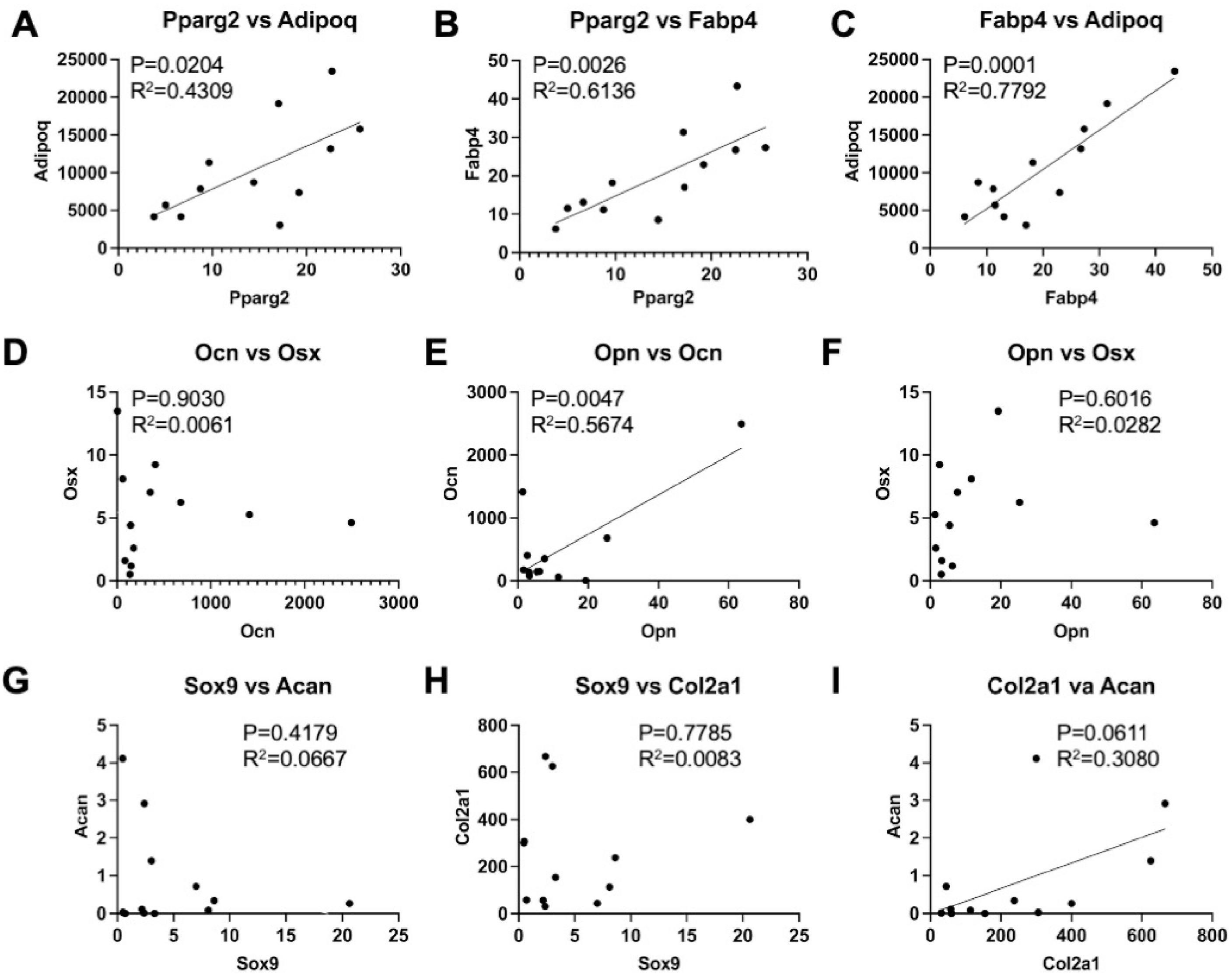


**Figure S5. Correlations among the expression levels of adipogenic marker genes (A-C), osteogenic marker genes (D-F) and chondrogenic marker genes (G-I) of differentiated DFAT-like cells of single-adipocyte origin.**
